# Arterial spin labeled MRI detects clinically relevant increases in myocardial blood flow with vasodilatation

**DOI:** 10.1186/1532-429X-13-S1-O94

**Published:** 2011-02-02

**Authors:** Zungho Zun, Padmini Varadarajan, Ramdas G Pai, Eric C Wong, Krishna S Nayak

**Affiliations:** 1 University of Southern California, Los Angeles, CA, USA; 2 Loma Linda University Medical Center, Loma Linda, CA, USA; 3 University of California, San Diego, La Jolla, CA, USA

## Objective

This study sought to demonstrate the potential for arterial spin labeling (ASL) to differentiate normal and ischemic myocardial segments based on increase in myocardial blood flow (MBF) with vasodilatation.

## Background

Myocardial ASL is a promising technique for the assessment of MBF because of the absence of contrast agents. Patients with end-stage renal disease cannot tolerate contrast agent, and therefore stand to potential benefit from myocardial ASL. MBF in healthy myocardium is known to increase by 4 times during vasodilator-induced stress, compared to at rest [1].

## Methods

**Figure 1:**
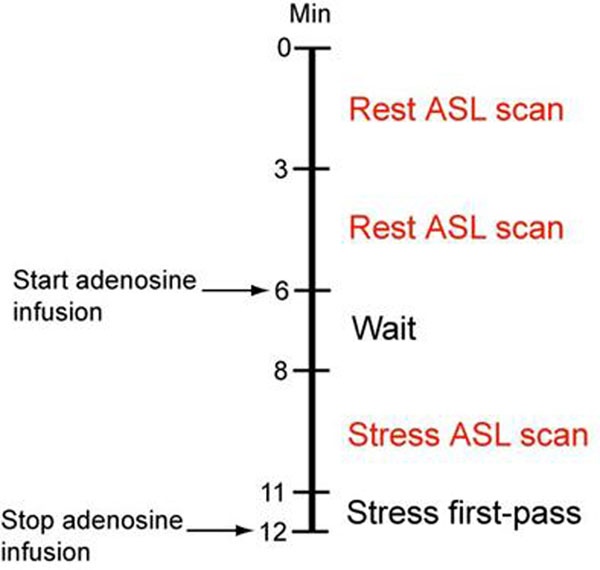
Imaging protocol excerpt.

Twenty nine patients were recruited from those scheduled for routine cardiac MR (CMR) exams. All MRI experiments were performed on a GE Signa 3T scanner. Myocardial ASL measurements were obtained from a single mid short-axis slice, using flow-sensitive alternating inversion recovery (FAIR) tagging and balanced steady-state free precession (SSFP) imaging [2]. Rest-stress myocardial ASL scans were incorporated in CMR exam including first-pass imaging during adenosine infusion of 0.14 mg/kg/min (Figure 1). Based on CMR results, patients who were suspected to have severe ischemic heart disease also underwent X-ray angiography.

## Results

Among 29 patients, fifteen patients were found to be normal based on having no visible perfusion defect on first-pass MRI and no significant stenosis on X-ray angiogram. Ten patients had both perfusion defects and stenosis. Four remaining patients showed perfusion defects but no stenosis. Table 1 summarizes the perfusion analysis performed in both whole myocardium and myocardial segments after excluding subjects with signal-to-physiological-noise ratio<2.0 [2]. The normal segments included all six segments [3] of the whole myocardium in normal patients and ischemic segments included the most ischemic segments in the patients with stenosis confirmed by X-ray angiography. MBF increase with adenosine in the global and segmental myocardium in normal patients were both statistically significant with p<0.0001 while MBF increase with adenosine in ischemic segments were not statistically significant with p=0.1032, based on paired t-test. Difference in perfusion reserve (MBF_stress_/MBF_rest_) between normal and ischemic segments was statistically significant with p=0.0296, based on unpaired t-test.

**Table 1:** MBF at rest and during stress (ml/g/min) and perfusion reserve.

Subject	Normal whole myocardium		Normal myocardial segments		Ischemic myocardial segments	
N	12		66		11	
Condition	Rest	Stress	Rest	Stress	Rest	Stress
MBF	1.19±0.46	3.99±1.39	1.20±0.88	3.90±1.30	1.48±0.46	2.17±1.53
Reserve	4.21±3.44		2.87±2.10		1.44±0.97	

## Conclusion

This study has demonstrated that myocardial ASL is able to capture adenosine-induced MBF increase in normal myocardium while detecting insignificant increase in ischemic myocardium. This suggests that myocardial ASL with vasodilation has a potential to diagnose angiographically significant heart disease.
